# Effect of Different Solutions on the Colour Stability of Nanoparticles or Fibre Reinforced PMMA

**DOI:** 10.3390/polym14081521

**Published:** 2022-04-08

**Authors:** Abdulaziz Alhotan, Alaaeldin Elraggal, Julian Yates, Julfikar Haider, Carlos Alberto Jurado, Nikolaos Silikas

**Affiliations:** 1 Division of Dentistry, School of Medical Sciences, University of Manchester, Manchester M13 9PL, UK; julian.yates@manchester.ac.uk (J.Y.); j.haider@mmu.ac.uk (J.H.); nikolaos.silikas@manchester.ac.uk (N.S.); 2 Dental Health Department, College of Applied Medical Sciences, King Saud University, Riyadh 11454, Saudi Arabia; 3 Conservative Dentistry Department, Faculty of Dentistry, Alexandria University, Alexandria 21568, Egypt; alaa.elraggal@alexu.edu.eg; 4 Department of Engineering, Manchester Metropolitan University, Manchester M1 5GD, UK; 5 Woody L. Hunt School of Dental Medicine, Texas Tech University Health Sciences Centre El Paso, 5001 El Paso Drive, El Paso, TX 79905, USA; carlos.jurado@ttuhsc.edu

**Keywords:** PMMA, ZrO_2_ nanoparticle, TiO_2_ nanoparticle, E-glass fibre, colour stability, Steradent™, coffee

## Abstract

This study aimed to evaluate the colour stability of polymethyl methacrylate (PMMA) denture base reinforced with ZrO_2_ nanoparticles, E-glass fibres, and TiO_2_ nanoparticles at various concentrations over 180-day storage in Steradent™ (STD) denture cleaner or coffee (CF). A total of 130 disc-shaped specimens were fabricated at various filler concentrations and divided into four main groups to measure the colour changes. Groups Z, T, and E consisted of PMMA reinforced with ZrO_2_ nanoparticles, TiO_2_ nanoparticles, or E-glass fibre, respectively, while Group C consisted of PMMA specimens without filler served as the control group (*n* = 10). The three reinforced groups were further subdivided according to the filler content (*n* = 10) added to the PMMA (1.5%, 3.0%, 5.0%, and 7.0% wt.%). Half of the specimens were stored in STD, while the other half was stored in CF for 180 days. A Minolta Chroma Meter was used to measure the colour changes (ΔE) at 7, 30, 90 and then 180 days. The results were assessed using two-way repeated-measures analysis of variance (RM-ANOVA) along with Bonferroni post hoc tests at a *p* ≤ 0.05 significance level. Significant different colour changes (ΔΕ) were observed between all tested groups and across different time points. TiO_2_-reinforced PMMA in STD/CF showed the lowest colour stability, while the E-glass fibre-reinforced PMMA in STD/CF showed the highest colour stability. Furthermore, coffee appeared to have the greatest impact on the colour change in comparison to the Steradent^TM^. The results indicated that the filler type and concentration, type of solution, and length of storage all affected the colour stability of the tested specimens.

## 1. Introduction

Polymethyl methacrylate (PMMA) has been successfully used for many years to construct partial or complete edentulous denture bases due to its excellent colour matching with the gum, cheap to fabricate, and biocompatibility within the oral environment [1,2]. One parameter that directly affects the long-term aesthetics of a dental material is colour stability [3,4,5,6]. The optical properties of colour and translucency directly influence the aesthetic appearance of a denture base material [7,8]. Translucency determines the extent to which light can pass through a material [7]. To ensure aesthetically pleasing results, it is important that denture base resin mimics the natural appearance of oral tissue and offers long-term colour stability [8,9]. When the denture material becomes discoloured or faded, patients may request a new denture [10,11,12]. Thus, it is important that the colour of the denture material remains consistent over time and can withstand conditions within the oral cavity [6,9,13].

PMMA denture bases are hydrophilic materials which could be discoloured when occasionally exposed to extrinsic stains [11,12] from the consumption of dark drinks (tea/coffee) or food containing artificial colouring [5,9,14]. According to Dietschi et al. [15], the water acts as a carrier of pigments and dyes of the stain. Thus, highly hydrophilic materials that exhibit high solubility are at an increased risk of staining in response to exposure to hydrophilic colourants in aqueous solutions [2,11].

The majority of patients clean their dentures using chemical agents, which are categorised according to their chemical composition, and these include disinfectants agents, enzymes, acids, and alkaline peroxides [9,16,17]. It is important that denture cleaners are selected in accordance with their compatibility with the denture base material [17]. Denture cleaners are frequently used to eliminate staining from the surface of denture bases and prohibit plaque formation [10]. However, daily use of these cleaners can have a negative effect on the properties of the base resin, leading to changes in its hardness, gloss, colour, or smoothness. [16]. Previous studies have determined that the type of denture cleaner used can have a direct impact on the colour changes observed. Furthermore, the extent of colour change observed positively correlates with the length of time the dentures are exposed to the cleaner [6,17,18]. In this regard, denture cleaners must be chosen with due consideration of the extent to which they are compatible with the denture base material [17].

Incorporation of various nanoparticles such as zirconium (ZrO_2_) and titanium (TiO_2_) have attracted a significant attention in recent times on the basis that they offer high chemical stability [19], optimum mechanical properties, resistance to wear, and non-toxicity [20,21]. Nanoparticles are characterised by high surface area/volume ratio, which gives the material different optical and mechanical properties [1]. Glass fibres such as E-glass also provide improved mechanical properties, favourable aesthetic qualities, biocompatibility, and stability in the oral environment [22,23]. The optical properties of PMMA are affected by the type, size, shape, and concentration of the incorporated fillers [24]. These filler materials represent a viable option for reinforcing denture acrylic resins, enhancing their mechanical properties. However, the effect of these filler loadings on colour stability of PMMA denture base needs to be further investigated. Therefore, the current study aimed to measure the effect of different concentrations of E-glass fibre, ZrO_2_, or TiO_2_ nanoparticles fillers on the colour stability of reinforced PMMA denture base resins after immersion in staining or cleaning solutions. The study was guided by the null hypothesis that incorporating different filler types and concentrations into PMMA acrylic resin would not affect the colour stability over 180 days of storage in denture cleanser or coffee.

## 2. Materials and Methods

### 2.1. Materials

Table 1 presents an overview of the commercial materials used in the study to evaluate the colour changes observed in the reinforced PMMA acrylic resin in response to exposure to staining fluids.

### 2.2. Sample Preparation

One-hundred-and-thirty disc-shaped samples (*n* = 10) of 25 ± 1 mm in diameter and 2.0 ± 0.2 mm in thickness were made using a brass mould. The mould was designed to produce specimens with slightly modified dimensions to those outlined in ISO:1567. ZrO_2_ and TiO_2_ nanoparticles and E-glass fibre were separately incorporated into the PMMA acrylic resin at four different weight concentrations: 1.5 wt.%, 3.0 wt.%, 5.0 wt.%, and 7.0 wt.%. The samples were split into four main groups: Group C (control group), and Group Z (ZrO_2_), Group T (TiO_2_), and Group E (E-glass fibre). The three filler material groups were further split into four subgroups relative to their wt.% of fillers as detailed above. Based on the available information in the literature, a pilot study was carried out to determine the weight concentrations of the fillers.

#### 2.2.1. Modification of Nano-ZrO_2_ and Nano-TiO_2_

To encourage the formation of a bond between the PMMA resin matrix and the inorganic filler particles, and create reactive groups, the surfaces of the PMMA particles were modified with 3 wt.% silane coupling agents (γ-MPS). A measure of 15 g of each filler type was separately dispersed into 70 mL of ethanol and agitated at a speed of 1500 rpm in a speed mixer (DAC 150.1 FVZK, High Wycombe, Buckinghamshire, UK) for 10 min. After speed mixing, γ-MPS (0.45 g, 3.0% wt) was added to each filler type and stirred in a magnetic stirrer at 200 rpm for 2 h at room temperature. Mixtures were refluxed for 240 min at 50 °C then centrifuged for 20 min at 4500 rpm at room temperature. The formed sediment was then dried in a Genevac machine (Genevac EZ-2 series, SP Scientific Company, Ipswich, UK) for 180 min at 50 °C leaving behind silanised fillers [23,25].

#### 2.2.2. Combining Fillers with PMMA/MMA

Each silanised filler material was individually incorporated into the MMA monomer at the required concentration to produce modified monomer (MMA monomer and filler). As per the manufacturer’s instructions, this modified monomer was consequently blended with PMMA powder at a ratio of 21 g/10 mL.

When the PMMA powder and modified monomer resembled a dough-like consistency, it was packed into a brass mould, which was then placed in a flask. The flask was then placed in a water bath curing unit at 23 °C to until the temperature reaches 95 °C polymerise the specimens. The specimens were extracted from the mould after completion of the polymerisation process and once the flask had cooled. These specimens were subsequently trimmed using a grinder before being polished with a range of SiC abrasive papers (600-, 800-, 1000- and 1200-grit) to smoothen the surface. The above procedure has been outlined in more depth in the authors’ previous papers [23,25]. Mechanical properties of the composites were also determined and presented the previous papers.

#### 2.2.3. Colour Measurement Process

To replicate the natural oral environment, the specimens from each group (*n* = 10) were stored individually in an incubator in distilled water at 37 °C for a 24 h period. A Minolta Chroma Meter CR-221 was then used to perform the baseline colour measurements for each tested group. After the baseline measurements had been taken, twenty specimens from each group were stored separately in either commercial denture cleaner or commercial coffee at 37 °C, over 180 days in darkened storage containers that replicated the oral conditions.

According to the manufacturer’s instructions, the coffee solution was produced by mixing 18 g of coffee powder with 200 mL of boiling water until dissolved and cooled to around 37 °C. The cleaning solution consisted of one Steradent™ denture-cleaning (3.184 g) tablet fully dissolved in 250 mL of distilled water.

The specimens in each tested group of specimens were separately immersed in coffee as a staining agent and Steradent™ as a cleaning solution. Both solutions were replenished daily. The total immersion duration of 180 days was equivalent to 15 years of oral exposure in the clinical setting [26].

The colourimetry measurements were taken for all the specimens in all tested groups at 7, 30, 90 and then 180 days after the start of the experiments. Before measurements were taken, the specimens were removed from their respective storage solution, washed with distilled water, and dried using tissue paper. All samples were then placed on the same white plate used to calibrate the Minolta Chroma Meter CR-221 instrument in line with the manufacturer’s guidelines.

All the colour change measurements (ΔE) were calculated using the Commission Internationale de l’Eclairage L*, a*, b* (CIE-Lab) colourimetric system, which is commonly employed to numerically classify colour differences. The device was carefully tuned to measure three colour dimensions: L* represents a range of lightness and darkness ranging from 0 (black) to 100 (white), while a* and b* represent chromaticity for red or green chroma: red generates a positive a*, green generates a negative a*, yellow generates a positive b*, and blue creates a negative b*. Throughout the measurement process, the specimens were placed in a lighting setup that was designed to simulate the standard daylight (CIE illuminant D:65), with a gauging region of 3 mm, a viewing angle of 0°, and an illumination of 45°.

Three readings per specimen were taken during each measurement stage before the L*, a*, and b* scales were calculated. The colour differences (ΔE) for the L*, a*, and b* values of all specimens were computed using the following equations:

∆E*ab = ([L*1−L*2]^2^ + [a*1−a*2]^2^ + [b*1−b*2]^2^)^2^ (1)

ΔΕ = [(ΔL*)^2^ + (Δa*)^2^ + (Δb*)^2^]^½^ (2)

Where L*1, a*1, and b*1 represent the baseline values; L*2, a*2, and b*2 represent the values following 180-day immersion; and ΔL*, Δa*, and Δb* represent the variations between the pre- and post-immersion values.

Based on the National Bureau of Standards (NBS) units for delineating colour differences, the CIE Lab measurements were used to ascertain the discernible colour change (ΔE*ab) for all the specimens (Table 2). These NBS unit values were determined using the following formula:

∆E*ab (in NBS unit) = ∆E*ab (in CIE) × 0.92(3)

### 2.3. Statistical Analysis

The colour change (ΔE) values were statistically assessed using a statistical software package (SPSS Statistics Version 25, IBM, New York, NY, USA). The outcomes of Shapiro–Wilk tests revealed that the *p*-values were not statistically significant, suggesting the data were normally distributed. The sphericity of the data was checked and verified using Mauchly’s test. The effect of different filler types and their relative percentages on the mean colour change values (ΔE) across different time points was statistically analysed using two-way repeated measures analysis of variance (RM-ANOVA) for each immersion solution. For all tests, a significance level of *p* ≤ 0.05 was set. A Bonferroni pairwise comparison test was subsequently conducted to ascertain the variations at the *p* ≤ 0.05 significance level. Additionally, colour difference (ΔE) data were computed per the National Bureau of Standards (NBS) units through the formula (ΔE × 0.92) to correlate the colour differences (ΔE) to the clinical environment.

## 3. Results

The mean and standard deviation (±SD) of the baseline colour measurement values (L*, a*, and b*) of the specimens in all the experimental groups following immersion in distilled water for 24 h are presented in Table 3. Table 4 and Table 5 present the standard deviation values, mean colour change values (ΔE), and colour expressed in NBS units at different time intervals for all experimental groups following immersion in the two solutions.

### 3.1. Steradent™ Solution

According to the Mauchly’s test of sphericity, no statistical significance (P = 0.55) was found; thus, sphericity was assumed. The main effect of time (within the subjects’ groups) on colour change scores was statistically significant, sphericity assumed; F = 222.7, P = 0.00. This effect, however, was qualified by a significant (time × materials) group interaction, sphericity assumed; F = 3.9, P = 0.00. This interaction indicated that the variation in the mean colour change (ΔE) over the repeated measurements occasion itself varied as a function of materials type membership. The main effect of different materials on the colour change across time (between subjects’ groups) was statistically significant F = 181.7, P = 0.00. As there was evidence of significant interaction between subjects and within subjects’ groups, it was mandatory to describe the nature of this interaction by further running simple-effects tests.

**Figure 1:**
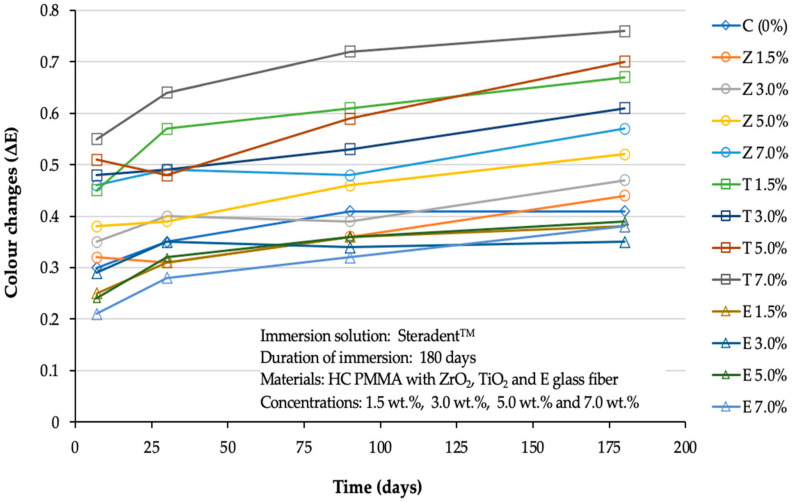
Line graph showing the cubic trend of the studied materials across the different time points in Steradent™ solution.

Irrespective of group membership, the mean change in colour followed a cubic trend across the four different time points with no statistical significance (P = 0.37) (Figure 1), however, the differential trend across different groups also followed a cubic trend with a statistical significance (P = 0.00), indicating that different materials had a different trend of colour change over time.

### 3.2. Coffee Solution

According to the Muachly test of sphericity, there was a statistical significance P = 0.00, thus sphericity was not assumed, and Greenhouse–Geisser correction was followed. The main effect of time (within the subjects’ groups) on colour change scores is statistically significant, based on Greenhouse–Geisser assumption (0.69); F = 1806.9, P = 0.00. This effect, however, is qualified by a significant (time × materials) group interaction, Greenhouse–Geisser assumed; F = 62.1, P = 0.00. The main effect of different materials on the colour change across time (between subjects’ groups) was statistically significant F = 817.8, P = 0.01.

**Figure 2:**
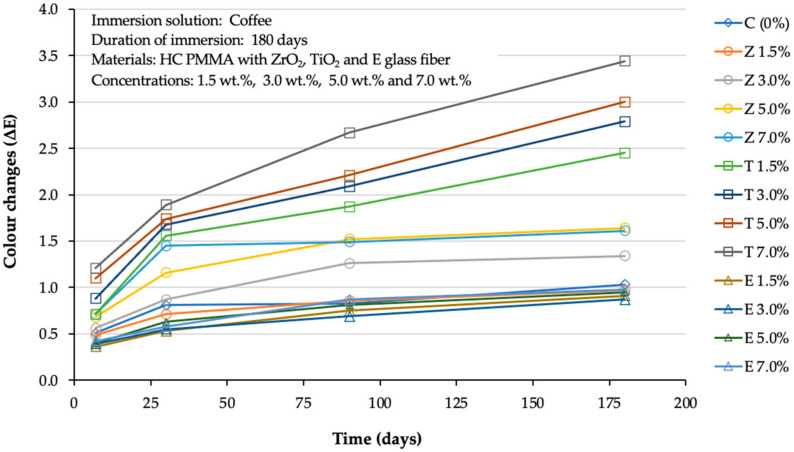
Line graph showing the cubic trend of the studied materials across the different time points in coffee solution.

Irrespective of group membership, the mean change in colour followed a cubic trend across the four different time points with a statistical significance (P = 0.01). Furthermore, the differential trend across different groups also followed a cubic trend with a statistical significance (P = 0.00) (Figure 2).

The colour change (ΔE) values observed in Group C ranged from 0.31to 0.41 for STD, and 0.51 to 1.03 for CF. The ΔE values of the ZrO_2_-reinforced groups ranged from 0.31 to 0.57 for STD, and from 0.49 to 1.64 for CF. The ΔE values of the TiO_2_ reinforced groups ranged from 0.45 to 0.76 for STD, and from 0.71 to 3.44 for CF. The ΔE values of the E-glass reinforced groups ΔE ranged from 0.21 to 0.39 for STD, and from 0.36 to 0.98 for CF.

**Figure 3:**
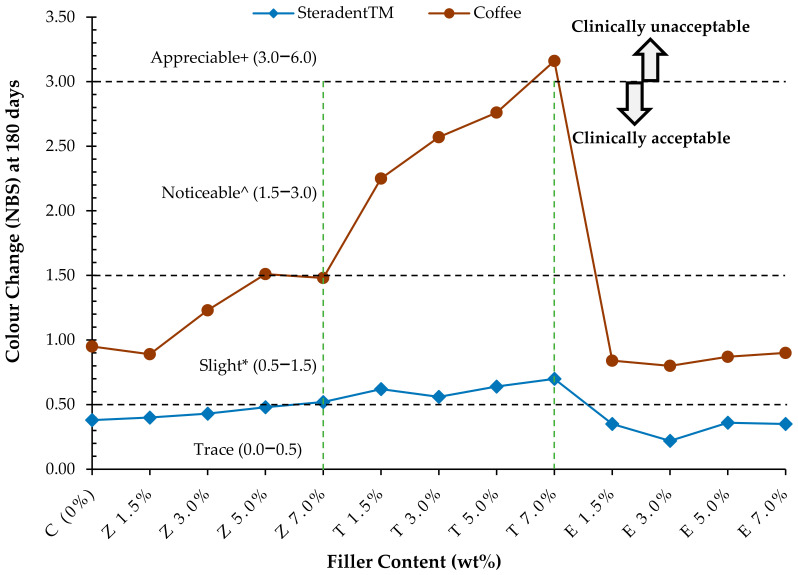
Line graph of the colour change observed (NBS unit system) in each group stored in Steradent™ and coffee for six months.

Table 6 and Figure 3 summarise the colour change values (ΔE) at six months expressed in NBS values. In the specimens stored in the CF solution, the largest colour change in CF (ΔE = 3.44; NBS = 3.16) was observed in the T7.0% group, while the smallest colour change (ΔE = 0.87; NBS = 0.80) was found in the E3.0% group. In the specimens stored in the STD solution, the largest colour change in STD (ΔE = 0.76; NBS = 0.70) was observed in the T7.0% group, while the smallest colour change (ΔE = 0.35; NBS = 0.22) was found in the E3.0% group.

All specimens in all groups immersed in the STD solution exhibited trace colour change with the exception of the Z7.0% and all T groups, which presented a slight colour change in comparison to Group C. When immersed in the CF solution, the specimens from the C, Z1.5%, Z3.0%, Z7.0% and all E groups exhibited slight colour changes, while those in the Z5.0%, T1.5%, T3.0% and T5.0% groups exhibited noticeable colour changes. Furthermore, an appreciable colour change was observed in specimens of the T7.0% group when immersed in the CF solution.

## 4. Discussion

Colours are determined and measured in the dental setting using two broad approaches: instrumental and visual [7,12]. Of these, instrumental colourimetric can represent a reliable means of preventing subjective errors arising during colour assessments [12,13]. A colourimeter was used in the current study to quantify the colour of each specimen using NBS units. The NBS parameter was selected because it plays an important role in quality control and colour comparison functions [18,27,28]. The outcomes of the current study revealed that there were statistically significant colour variations between the specimens in the groups reinforced with fillers in various concentrations and the control group to varying extents after being immersed in staining and cleaning liquids for pre-determined periods (Table 4 and Table 5). Thus, the null hypothesis was rejected.

In our previous study, the optimal filler concentrations for the flexural strength were found between 3.0–5.0% of ZrO_2_, 1.5% of TiO_2_, and 3.0–7.0% of E-glass fibre. On the other hand, for all the composites within this study, a filler concertation of 3.0 wt.% and above would significantly improve hardness [24]. It was also found that 1.5 wt.% and 3.0 wt.% of ZrO_2_; 1.5 wt.% of TiO_2_; and 1.5 wt.%, 3.0 wt.%, 5.0 wt.%, and 7.0 wt.% of E-glass fibre can effectively enhance the fracture toughness of PMMA. The inclusion of E-glass fibres did significantly improve the impact strength, while ZrO_2_ or TiO_2_ nanoparticles did not cause any significant change [25].

Colourimetric instruments or spectrophotometric techniques are frequently used to determine the colour of acrylic resins and other dental materials [11,13]. Photometric and colourimetric techniques are employed to measure and express colour using three coordinate values: L*, a*, b*. These techniques pinpoint the colour of the object within the two systems: The Munsell colour system and the standard Commission International de l’Eclairage (CIE L*a*b*) colour system [18].

To the best of the author’s knowledge, there are currently no alternative studies of this nature available in the literature against which these outcomes can be compared. However, the results of this study were aligned with those of Saleh et al. [14], who evaluated the colour stability of high-impact heat-polymerised denture base acrylic filled with ZrO_2_ nanoparticles. In their study, following storage in distilled water and two different denture cleaners for six months, significant colour differences (ΔΕ) were observed between all the groups immersed in denture cleaners. Furthermore, these values increased over time [14]. The findings of the current study were also in agreement with those of Tuncdemir et al. [5], who determined that the use of glass and polyethylene fibre reinforcement and composite resin led to colour changes after accelerated aging. The variations observed in the outcomes of the existing studies may be attributed to variations in the colour and form of the composite materials used, the type of denture cleaning agents used, the nature of the filler employed, or variations in the polymerisation type [5,16,18]. The quality of the pigment, resin matrix type, filler (size and content) and other chemical additives also have a significant role to play in any colour change that may be observed in composite resins [5]. For instance, composite resins that are impregnated with fillers of large particle size could be more vulnerable to discolouration as a result of water aging [15,29].

In the current study, the colour changes observed among the majority of the experimental groups typically increased in line with an increase in the storage time (Figure 1 and Figure 2). However, the extent to which the solution involved influenced the colour changes differed according to the material group. Furthermore, all groups exhibited a lower degree of colour change when stored in denture cleanser in comparison to coffee. According to the dental clinical criteria proposed (NBS), a ∆E below 1.0 unit cannot be detected by the human eye. Generally, ∆E values between 1.0 and 3.20 units are clinically acceptable, while those above 3.30 units are not clinically acceptable [5,9,30].

Acrylic resin typically absorbs liquids, such as water, because of the polarity of the PMMA molecules [13]. Following absorption, the solution disperses into the polymer network, thereby causing hydrolysis and the development of acrylic areas that exhibit aberrant optical properties which go onto cause the colour change [31]. According to the findings of a previous study, the colour changes of denture base materials may result from modifications to the material matrix and the staining mechanisms of external colourants [5,11,15]. In addition, leakage, water sorption, solubility, chemical degradation, and surface roughness can contribute to colour changes [3,6,27]. Other research has concluded that denture cleansers can effectively bleach or whiten the acrylic resin materials [6,32]. This may explain why the L*, a*, and b* values decreased or increased.

After storage in denture cleaner the colour changes of all tested groups (ΔE ≤ 0.76) were not detectable by the human eye; thus, they were considered clinically acceptable. However, the colour changes observed in the specimens stored in coffee did vary between the tested groups. A clinically acceptable or negligible colour change (ΔE ≤ 0.98) that was not detectable by the human eye was found in groups Z1.5% and E, while the colour changes observed in groups C, Z3.0%, Z5.0%, Z7.0%, T1.5%, T3.0% and T5.0% were visually noticeable (ΔE ≤ 3.00) but clinically acceptable or negligible colour change. The only colour change that was not clinically acceptable was group T7.0% (ΔE ≤ 3.44) because it exceeded the threshold ΔE value.

The resin composites change in multiple ways in response to the polymerisation reaction and due to the interaction with chemicals from food and drinks and the moist oral environment [2,7,9]. This physical transformation could potentially result in the resin matrix softening and lead to a reduction in colour stability [33]. In general, the variation in colour change values could be attributed to the background colour, measuring instrument, sample size, and illumination [5,34]. In this study, these factors remained constant throughout all measurements and would have had negligible impact on the relative values of the colour changes. The colour changes may be the result of other factors, including storage conditions and time periods, filler concentrations, and the resin composite itself [6,35].

Colour stability and gloss play a significant role in the physical appearance of denture bases, and any changes to these characteristics may provide an indication of the way in which the denture base material will degrade [36]. The increase in the colour change (ΔE) values is suggestive of a surface gloss loss [17,37]. A study has found that a glossy, smooth surface prevents discolouring films from forming, impedes plaque formation, and assists the removal of plaque [17]. The structure of the acrylic resin in combination with the accumulation of substances can also impact the colour stability, particularly if the material has a higher surface roughness [8,38]. Polychronakis et al. [36] assessed the impact that denture cleaners had on the surface roughness, colour and gloss of nylon and heat-polymerised acrylic denture base resins before and after immersion in test liquids for 30 days. Their findings revealed that gloss, surface roughness and colour were altered in both denture types [36]. Furthermore, the water temperature in which the solutions are prepared represents a fundamental factor, resulting in the acrylic resin becoming whiter when patients use hot water [39,40]. Devlin and Kaushik found that using a hot alkaline peroxide solution led to water absorption on acrylic surfaces, resulting in permanent surface whitening after the specimens were dried [39].

In the current study, the T7.0% group stored in coffee solution for six months presented a colour change unit that exceeded the clinically acceptable threshold (ΔE > 3.30). Thus, it was concluded that immersion of denture base resin in coloured beverages over a certain period resulted in discolouration; however, the extent of the discolouration varied across the groups in the different storage mediums. Hong et al. [18] found that there was a positive correlation between colour change in acrylic resins and immersion time as a result of monomer leaching out and the absorption of water. Furthermore, Goiato et al. [38] concluded that any monomer released during storage could interact with the glaze layer, resulting in increased colour change. The primary discolouring mechanism is likely to be the sorption of liquids (solutions) [9]. Researchers have documented a relationship between water sorption and hygroscopic expansion, and denture base staining [31,40]. There is a high probability that denture base resins that absorb water will also absorb any other forms of liquids, including those that contain staining agents [31]. After a denture base resin has absorbed water, its polymer matrix expands, leading to the polymer chains becoming separated such that staining agents can subsequently penetrate and stain the denture base material [9].

In the current study, a more significant level of colour change was observed in the specimens that were stored in coffee solution when compared to those stored in STD. This finding was in agreement with that of Buyukyilmaz and Ruyter [41], who concluded that coffee generated a higher degree of staining than any other solution. Coffee has also been found to greatly increase the extent to which dental materials are discoloured, and the discolouration of polymeric materials as a result of prolonged exposure to coffee has been linked with surface sorption [11,13]. It is considered that the tannic acid (pH 6–6.4) that is contained within coffee is responsible for its yellow-brown colour; thus, staining is commonly attributed to this ingredient [12,42]. Resins become discoloured after exposure to coffee because of the absorption and adsorption of the various yellow colourants [12,42].

The STD employed in the current study took the form of a 115.1-mg peroxide tablet that had a pH value of 11 [18]. It has been reported that denture cleaners cause the plasticisers and any soluble components to leach out of the denture base resin [40]. In addition, colour change results from the absorption of water and any other salivary elements into the resin matrix [6,40]. Peroxide-based denture cleansers incorporate an effervescent element, such as sodium bicarbonate or sodium perborate [18,40]. After tablets are dissolved, the sodium perborate breaks down to produce an alkaline peroxide solution that consequently releases oxygen, which loosens debris [14,32]. Thus, denture cleansers can lead to hydrolysis and the degradation of the polymerised acrylic resin [18]. Some researchers have reported that denture cleaners may whiten or bleach denture acrylic resins after a period of storage, while water absorption also has an impact on their physical properties [32,40]. Water acts as a solvent or pigment carrier; thus, decreasing the amount of water that is absorbed into the material can concurrently decrease pigment penetration [11,15].

There is a positive correlation between resin degradation and the absorption/solubility of the base resin. The lower the level of water absorption/solubility, the lower the degradation, further reducing the solubility and water absorption [11]. The matrix/filler interface degrades as a result of water absorption. The water solution readily diffuses throughout the resin matrix, leading to swelling and tensile stress formation at the interface of the matrix and filler, which consequently enhances leaching and diffusion [15,31]. Following degradation of the resin composites, gaps or micro-passageways develop in the matrix/filler interface through which stains can easily penetrate thereby reducing the mechanical properties of the material [5,15]. This was evidenced in our previous SEM images of the fractured surface of the PMMA/E-glass fibres specimens, which revealed very little or no gaps around the fibres and thus indicated a good adhesion between the PMMA matrix and the fibres. Furthermore, the SEM images of the fractured surface of the PMMA/ZrO_2_ nanoparticle specimens presented signs of particle clustering, while large pores in the TiO_2_ nanoparticle specimens indicated that a greater space in between the matrix discontinued the stress distribution. This could be attributed to the non-homogeneous distribution or agglomeration particles in the matrix serving to weaken the physical and mechanical performance of the PMMA/TiO_2_ nanoparticles.

The outcomes of the two-way RM-ANOVA tests (Table 4 and Table 5) confirmed that all tested groups exhibited some degree of discolouration following immersion in the two media. However, the extent of the discolouration varied according to the type of medium. In Group C, the two solutions appeared to have different effects on the colour stability, with the extent of discolouration differing according to immersion times. Within ZrO_2—_and E-glass-reinforced groups immersed in STD solutions over six months exhibited a comparable level of colour change; however, those immersed in the CF solution exhibited the highest degree of colour change. Within TiO_2_-reinforced groups immersed in the solutions for over six months, the two solutions had different impacts on the colour change in most of the tested groups. The most severe colour change was observed in the specimens immersed in CF, followed by STD. It should be noted that in the case of Steradent, the trend of reaching colour saturation was similar for all the composites after 180 days, as seen in Figure 1. On the other hand, with coffee, ZrO_2_ and e-glass based composites reached colour saturation quite quickly. However, for the TiO_2_-based composites, the colour change was still continuing after 180 days, and this highlighted rather poor colour stability characteristics of the TiO_2_-based composites.

To surmount the limitations of the current study, it would be useful for future studies to assess gloss for PMMA filler-composite denture bases. Natural biocompatible colour pigments can be incorporated into the PMMA denture base material to overcome the colour change in PMMA that results from the incorporation of nanoparticles/fibres. Furthermore, the number of specimens included in each group should be increased to generate a superior statistical distribution. Mechanical properties of the aged specimens could be determined to assess the effects of aging.

The PMMA filler-composite denture bases impregnated with up to 7.0 wt.% of ZrO_2_, 5.0 wt.% of TiO_2_ and 7.0 wt.% E-glass fibre can sustain clinically acceptable colour stability over a prolonged period when stored in coffee, or denture cleaner. Thus, these filler composites percentages represent a viable replacement to traditional PMMA while also increasing the clinical lifespan of the synthetic denture.

## 5. Conclusions

This study found that the type of filler materials, storage time, and immersing solutions have a direct impact on the colour stability of the PMMA denture base resin. Most tested groups increased colour values as immersion times increased. Reinforcing the PMMA resin with nanoparticles/fibres did lead to colour changes that were both above and below the clinically acceptable standards depending on the filler type and the media employed for testing. When assessed in a line with the NBS unit, the colour changes observed in all groups following storage in all media were clinically acceptable, with the exception of the T7.0% group immersed in CF, which was clinically unacceptable. The findings also revealed that incorporating E-glass fibre into PMMA achieved the most stable colour, followed by ZrO_2_, and TiO_2_. Thus, incorporating fibre or nanoparticles into PMMA denture bases can help to produce dentures that can withstand stains and achieve longer clinical service. STD or CF solutions discoloured the materials in a time-dependent way through different mechanisms; thus, the extent of the staining differs. Coffee had a more significant negative impact on the colour stability of the tested specimens compared to the STD.

## Figures and Tables

**Table 1:** Materials utilised in the current study.

Material	Composition and Specifications	Manufacturer
Lucitone-199™	Heat-polymerised acrylic resin powder: PMMA; monomer: MMA	Dentsply International, York, PA, USA
Zirconium oxide	Zirconium (IV) oxide-yttria stabilised, nanopowder, <100 nm particle size	Sigma Aldrich, Gillingham, UK
Titanium oxide	Titanium (IV) oxide, anatase, nanopowder, <25 nm particle size	Sigma Aldrich, Gillingham, UK
Silanised E-glass fibre	3 mm in length, 15 μm in diameter	Hebei Yuniu Fibreglass, Xingtai, China
Ethanol	Ethanol, absolute (C_2_H_6_O, EtOH)	Fisher Scientific, Loughborough, UK
Silane coupling agent	3-(Trimethoxysilyl) propyl methacrylate, assay 98%	Sigma Aldrich, Gillingham, UK
Steradent™	Sodium bicarbonate, potassium carbonate peroxide, sodium sulfate, citric acid	Reckitt Benckiser Healthcare, Dansom Lane, Limited, UK
Nescafe original instant coffee™	Arabica and Robusta coffee beans	Nestle Ltd., York, UK

**Table 2:** Critical levels of colour changes in line with NBS System.

Critical Levels of Colour Changes	∆E NBS Units
Trace	0.0–0.5
Slight	0.5–1.5
Noticeable	1.5–3.0
Appreciable	3.0–6.0
Much	6.0–12.0
Very much	12.0+

**Table 3:** Mean (±SD) of baseline CIELab colour measurements for each tested group after immersion in distilled water for 24 h.

Tested Groups	L* Mean (SD)	a* Mean (SD)	b* Mean (SD)
C (0%)	6.02 (0.76)	0.32 (0.00)	0.31 (0.00)
Z 1.5%	10.14 (0.20)	0.34 (0.00)	0.33 (0.00)
Z 3.0%	15.61 (0.46)	0.35 (0.00)	0.34 (0.00)
Z 5.0%	20.86 (2.28)	0.35 (0.00)	0.34 (0.00)
Z 7.0%	25.61 (0.39)	0.36 (0.00)	0.35 (0.00)
T 1.5%	19.80 (1.55)	0.35 (0.00)	0.35 (0.00)
T 3.0%	24.90 (1.98)	0.36 (0.00)	0.36 (0.00)
T 5.0%	35.73 (1.42)	0.37 (0.00)	0.37 (0.00)
T 7.0%	38.80 (2.31)	0.37 (0.00)	0.38 (0.00)
E 1.5%	8.49 (4.5)	0.33 (0.00)	0.32 (0.00)
E 3.0%	6.22 (0.23)	0.33 (0.00)	0.31 (0.00)
E 5.0%	6.41 (0.60)	0.33 (0.00)	0.31 (0.00)
E 7.0%	6.10 (0.32)	0.33 (0.00)	0.31 (0.00)

**Table 4:** Results of the two-way RM-ANOVA with descriptive statistics of (ΔE) and National Bureau of Standards (NBS) unit at four different time intervals for each group after immersion in Steradent™.

Tested Groups	Steradent™			
	Day 1 ΔE (SD) NBS	Day 30 ΔE (SD) NBS	Day 90 ΔE (SD) NBS	Day 180 ΔE (SD) NBS
C (0%)	0.30 ^Aa^ (0.02) 0.29	0.35 ^Aab^ (0.03) 0.32	0.41 ^Bab^ (0.03) 0.38	0.41 ^Ba^ (0.04) 0.38
Z 1.5%	0.32 ^Aa^ (0.03) 0.29	0.31 ^Aa^ (0.02) 0.30	0.36 ^Aa^ (0.03) 0.33	0.44 ^Ba^ (0.03) 0.40
Z 3.0%	0.35 ^Aa^ (0.03) 0.32	0.40 ^Ab^ (0.03) 0.37	0.39 ^Aa^ (0.03) 0.36	0.47 ^Ba^ (0.02) 0.43
Z 5.0%	0.38 ^Aa^ (0.04) 0.35	0.39 ^Ab^ (0.02) 0.36	0.46 ^Bb^ (0.03) 0.42	0.52 ^Cb^ (0.04) 0.48
Z 7.0%	0.46 ^Ab^ (0.10) 0.42	0.49 ^Ac^ (0.02) 0.45	0.48 ^Abc^ (0.02) 0.44	0.57 ^Bb^ (0.05) 0.52
T 1.5%	0.45 ^Abc^ (0.05) 0.41	0.57 ^Bd^ (0.04) 0.52	0.61 ^BCd^ (0.03) 0.56	0.67 ^Cc^ (0.05) 0.62
T 3.0%	0.48 ^Abc^ (0.08) 0.44	0.49 ^ABc^ (0.03) 0.45	0.53 ^Bce^ (0.03) 0.49	0.61 ^Cbc^ (0.03) 0.56
T 5.0%	0.51 ^Abc^ (0.04) 0.47	0.48 ^Ac^ (0.05) 0.44	0.59 ^Be^ (0.05) 0.54	0.70 ^Cd^ (0.05) 0.64
T 7.0%	0.55 ^Ac^ (0.04) 0.51	0.64 ^Bd^ (0.06) 0.59	0.72 ^Cf^ (0.04) 0.66	0.76 ^Cd^ (0.03) 0.70
E 1.5%	0.25 ^Aad^ (0.03) 0.23	0.31 ^ABa^ (0.04) 0.30	0.36 ^BCa^ (0.05) 0.33	0.38 ^Ca^ (0.06) 0.35
E 3.0%	0.29 ^Aad^ (0.02) 0.27	0.35 ^Aab^ (0.03) 0.32	0.34 ^Aa^ (0.03) 0.31	0.35 ^Aa^ (0.05) 0.22
E 5.0%	0.24 ^Aad^ (0.01) 0.22	0.32 ^ABa^ (0.02) 0.29	0.36 ^BCa^ (0.05) 0.33	0.39 ^Ca^ (0.02) 0.36
E 7.0%	0.21 ^Ad^ (0.01) 0.19	0.28 ^ABa^ (0.03) 0.26	0.32 ^BCa^ (0.03) 0.29	0.38 ^Ca^ (0.03) 0.35

Similar superscript capital letters in the same row indicate no significant difference (*p* > 0.05) within a group at different periods. Similar superscript small letters in the same column indicate no significant difference between tested groups (*p* > 0.05).

**Table 5:** Results of the two-way RM-ANOVA with descriptive statistics of (ΔE) and National Bureau of Standards (NBS) unit at four different time intervals for each group after immersion in coffee.

Tested Groups	Coffee			
	Day 7 ΔE (SD) NBS	Day 30 ΔE (SD) NBS	Day 90 ΔE (SD) NBS	Day 180 ΔE (SD) NBS
C (0%)	0.51 ^Aa^ (0.03) 0.47	0.81 ^Ba^ (0.02) 0.74	0.83 ^BCa^ (0.06) 0.76	1.03 ^Ca^ (0.09) 0.95
Z 1.5%	0.49 ^Aa^ (0.02) 0.45	0.71 ^Ba^ (0.06) 0.65	0.86 ^Ca^ (0.04) 0.79	0.97 ^Ca^ (0.05) 0.89
Z 3.0%	0.56 ^Aa^ (0.02) 0.52	0.87 ^Ba^ (0.05) 0.8	1.26 ^Cb^ (0.17) 1.16	1.34 ^Ca^ (0.13) 1.23
Z 5.0%	0.68 ^Ab^ (0.05) 0.63	1.16 ^Bb^ (0.11) 1.07	1.52 ^Cc^ (0.07) 1.40	1.64 ^Cb^ (0.06) 1.51
Z 7.0%	0.71 ^Ab^ (0.05) 0.65	1.45 ^Bc^ (0.04) 1.33	1.49 ^Bc^ (0.07) 1.37	1.61 ^Bb^ (0.06) 1.48
T 1.5%	0.71 ^Ab^ (0.03) 0.65	1.56 ^Bcd^ (0.11) 1.44	1.87 ^Cd^ (0.09) 1.72	2.45 ^Dc^ (0.12) 2.25
T 3.0%	0.88 ^Ac^ (0.12) 0.81	1.68 ^Bde^ (0.08) 1.55	2.09 ^Ce^ (0.17) 1.92	2.79 ^Dd^ (0.18) 2.57
T 5.0%	1.10 ^Ad^ (0.06) 1.01	1.74 ^Be^ (0.08) 1.60	2.21 ^Ce^ (0.11) 2.03	3.00 ^Dd^ (0.19) 2.76
T 7.0%	1.21 ^Ad^ (0.18) 1.11	1.89 ^Bf^ (0.08) 1.74	2.67 ^Cf^ (0.12) 2.46	3.44 ^De^ (0.22) 3.16
E 1.5%	0.36 ^Ae^ (0.02) 0.33	0.53 ^Bg^ (0.06) 0.17	0.75 ^Cag^ (0.05) 0.69	0.91 ^Caf^ (0.06) 0.84
E 3.0%	0.39 ^Ae^ (0.03) 0.36	0.55 ^Bg^ (0.03) 0.49	0.69 ^Cg^ (0.04) 0.63	0.87 ^Cf^ (0.06) 0.80
E 5.0%	0.40 ^Aae^ (0.03) 0.37	0.63 ^Bg^ (0.03) 0.58	0.81 ^Cag^ (0.02) 0.75	0.95 ^Caf^ (0.04) 0.87
E 7.0%	0.42 ^Aae^ (0.02) 0.39	0.58 ^Bg^ (0.03) 0.53	0.87 ^Cag^ (0.03) 0.80	0.98 ^Caf^ (0.08) 0.90

Similar superscript small letters in the same row indicate no significant difference (*p* > 0.05) within a group at different periods. Similar superscript capital letters in the same column indicate no significant difference between tested groups (*p* > 0.05).

**Table 6:** Colour change values according to the NBS unit system after storing the specimens in different solutions for 180 days.

Tested Groups	NBS Values after 180 Days in Solutions	
	Steradent™	Coffee
C (0%)	0.38	0.95 *
Z 1.5%	0.40	0.89 *
Z 3.0%	0.43	1.23 *
Z 5.0%	0.48	1.51 ^
Z 7.0%	0.52 *	1.48 *
T 1.5%	0.62 *	2.25 ^
T 3.0%	0.56 *	2.57 ^
T 5.0%	0.64 *	2.76^
T 7.0%	0.70 *	3.16+
E 1.5%	0.35	0.84 *
E 3.0%	0.22	0.80 *
E 5.0%	0.36	0.87 *
E 7.0%	0.35	0.90 *

Critical marks of colour difference: trace (0.0–0.5); slight * (0.5–1.5); noticeable ^ (1.5–3.0) and appreciable+ (3.0–6.0).

## Data Availability

The data presented in this study are available within the article.
